# Genome-wide RNAi screen in *Drosophila* reveals Enok as a novel trithorax group regulator

**DOI:** 10.1186/s13072-019-0301-x

**Published:** 2019-09-23

**Authors:** Zain Umer, Jawad Akhtar, Muhammad Haider Farooq Khan, Najma Shaheen, Muhammad Abdul Haseeb, Khalida Mazhar, Aziz Mithani, Saima Anwar, Muhammad Tariq

**Affiliations:** 1 Syed Babar Ali School of Science and Engineering, Lahore University of Management Sciences, Lahore, 54792 Pakistan; 2 Present Address: Biomedical Engineering Centre, University of Engineering and Technology Lahore, KSK Campus, Lahore, Pakistan

**Keywords:** Epigenetic cellular memory, Gene regulation, trithorax group, Polycomb group, Enok, Genome-wide RNAi screen, Histone modifications, H3K23 acetyl transferase

## Abstract

**Background:**

Polycomb group (PcG) and trithorax group (trxG) proteins contribute to the specialization of cell types by maintaining differential gene expression patterns. Initially discovered as positive regulators of HOX genes in forward genetic screens, trxG counteracts PcG-mediated repression of cell type-specific genes. Despite decades of extensive analysis, molecular understanding of trxG action and regulation are still punctuated by many unknowns. This study aimed at discovering novel factors that elicit an anti-silencing effect to facilitate trxG-mediated gene activation.

**Results:**

We have developed a cell-based reporter system and performed a genome-wide RNAi screen to discover novel factors involved in trxG-mediated gene regulation in *Drosophila*. We identified more than 200 genes affecting the reporter in a manner similar to trxG genes. From the list of top candidates, we have characterized Enoki mushroom (Enok), a known histone acetyltransferase, as an important regulator of trxG in *Drosophila*. Mutants of *enok* strongly suppressed extra sex comb phenotype of *Pc* mutants and enhanced homeotic transformations associated with *trx* mutations. Enok colocalizes with both TRX and PC at chromatin. Moreover, depletion of Enok specifically resulted in an increased enrichment of PC and consequently silencing of trxG targets. This downregulation of trxG targets was also accompanied by a decreased occupancy of RNA-Pol-II in the gene body, correlating with an increased stalling at the transcription start sites of these genes. We propose that Enok facilitates trxG-mediated maintenance of gene activation by specifically counteracting PcG-mediated repression.

**Conclusion:**

Our ex vivo approach led to identification of new trxG candidate genes that warrant further investigation. Presence of chromatin modifiers as well as known members of trxG and their interactors in the genome-wide RNAi screen validated our reverse genetics approach. Genetic and molecular characterization of Enok revealed a hitherto unknown interplay between Enok and PcG/trxG system. We conclude that histone acetylation by Enok positively impacts the maintenance of trxG-regulated gene activation by inhibiting PRC1-mediated transcriptional repression.

## Background

In multicellular eukaryotes, specialization of cell types is initiated by the onset of differential gene expression patterns in response to specific signals during early development. These differential gene expression profiles contribute to cell fate determination and differentiation during subsequent development. Thus, cell type-specific gene expression patterns are transmitted through cell divisions to daughter cells by a process known as epigenetic transcriptional cellular memory. In plants and mammals, DNA methylation, together with specific covalent modifications of histones, ensures faithful inheritance of cell type-specific gene expression patterns [1–3]. Genetic analyses in *Drosophila* discovered two groups of genes, the Polycomb Group (PcG) and the trithorax Group (trxG), that contribute to the maintenance of cellular memory [4–8]. The PcG maintains genes in a repressed state whereas trxG proteins act as anti-silencing factors and ensure activation of cell type-specific genes. Proteins encoded by the PcG and trxG genes act in different multiprotein complexes and modify local properties of chromatin to maintain transcriptional repression or activation of their target genes, respectively [9]. The PcG complexes, Polycomb Repressive Complex 1 and 2 (PRC1 and PRC2), are linked to histone H2A lysine 118 mono-ubiquitination (H2AK118ub1) [10] and histone H3 lysine 27 trimethylation (H3K27me3) [11–14], respectively, to maintain heritable patterns of repression. In contrast, different trxG complexes are known to deposit histone H3 lysine 4 trimethylation (H3K4me3) [15] and histone H3 lysine 27 acetylation (H3K27ac) [16], known hallmarks of active gene expression. Besides histone-modifying proteins, trxG also includes ATP-dependent chromatin remodeling factors that achieve an open conformation of DNA to allow transcription [9]. Molecular and biochemical characterization has revealed that the heterogeneous group of trxG proteins not only contributes to epigenetic cellular memory but also plays a role in general transcriptional activation [8].

In *Drosophila*, PcG/trxG proteins bind to specialized *cis*-acting elements called *PREs*/*TREs* (*Polycomb Response Elements*/*Trithorax Response Elements*) [17, 18] to maintain transcriptional cellular memory [19]. Several *PRE*/*TRE* elements exist within homeotic gene clusters (i.e., Bithorax Complex and Antennapedia Complex) and non-homeotic targets of PcG/trxG. High-resolution mapping of PcG-binding sites in *Drosophila* has identified hundreds of *PREs* genome wide [20–22]. In addition, several *PREs* have been shown to maintain stable and heritable gene expression of reporter genes in transgene reporter assays. Transgenic flies carrying either *iab*-*7PRE* or *bxd*-*PRE* fused to reporter genes have been extensively used to characterize mitotic and meiotic inheritance of PcG/trxG-dependent cellular memory [19].

Initially, trxG genes were identified as positive regulators of HOX genes in forward genetic screens. Numerous other trxG members were identified as suppressors of PcG-dependent homeotic phenotypes or as mutations that mimic loss of function of HOX genes in *Drosophila* [8]. Here, we have developed a cell-based reporter assay which is sensitive to the changing levels of PcG and trxG. This reporter was used to perform a large-scale genome-wide RNAi screen to discover new trxG genes using *Drosophila* cell culture. Employing stringent criterion, more than 200 genes were identified as potential trxG regulators, including known members of trxG and chromatin modifiers. Using a range of in vitro and in vivo assays, we have validated *Drosophila* Enok as a trxG regulator that strongly suppresses *Pc* mutant phenotype and enhances *trx* mutant phenotype. Further, we show that Enok colocalizes with TRX and its depletion results in enhanced levels of PC at chromatin and consequent downregulation of trxG target genes. Moreover, reduced expression of trxG targets after depletion of Enok correlates with an increased mono-ubiquitination of histone H2A at lysine 118 (H2AK118ub1) and an increase in stalled RNA Polymerase II. Our results suggest that Enok counteracts PcG-mediated repression and influences the ability of trxG to maintain transcription of its target genes.

## Results

### A cell-based reporter system for RNAi screen to discover novel trxG regulators

**Fig. 1:**
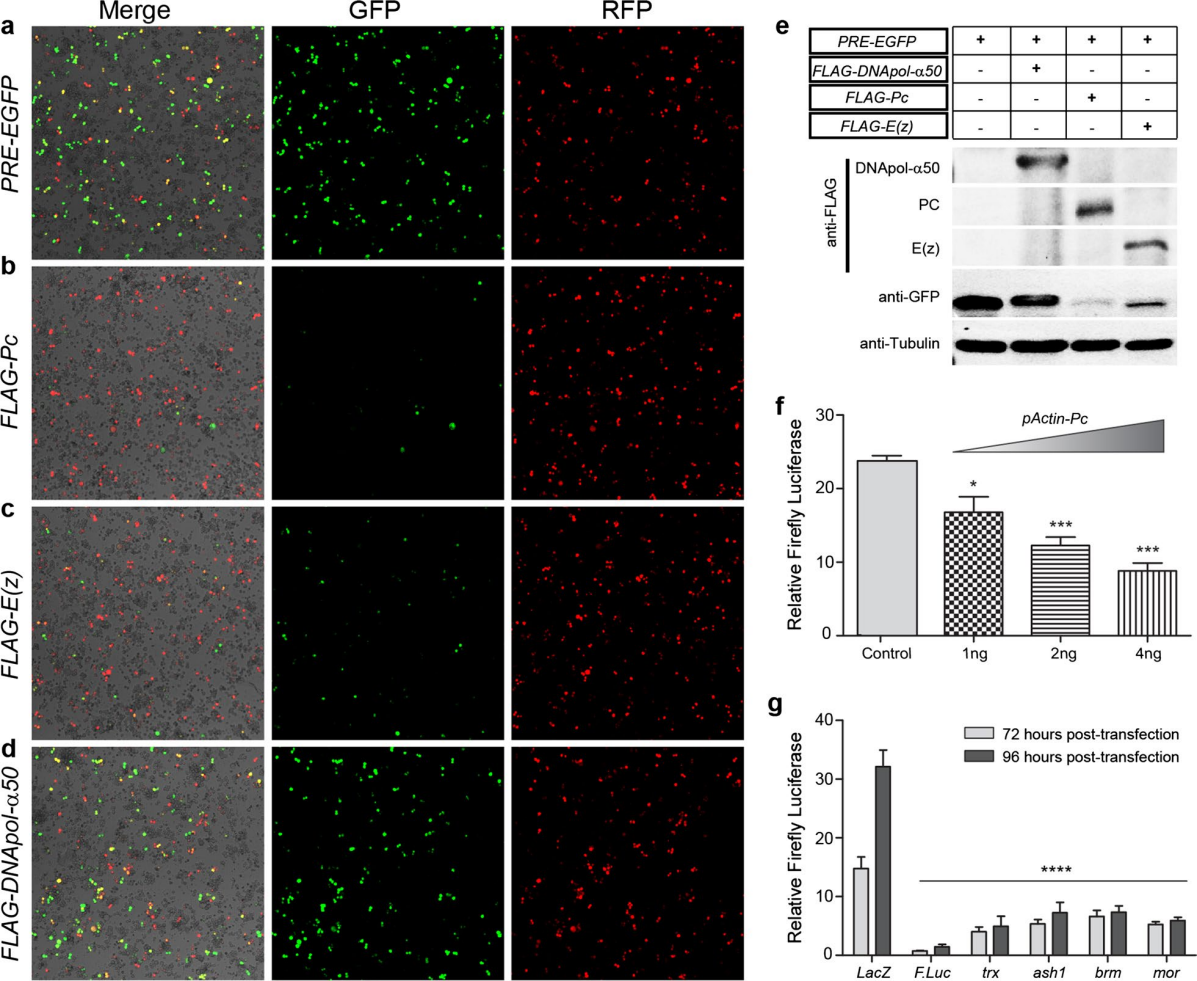
Proof of concept for PcG/trxG-mediated regulation of *PRE*-*EGFP* and *PRE*-*F.Luc* reporters. **a** *Drosophila* Schneider (S2) cells co-transfected with *PRE*-*EGFP* and *pActin*-*RFP* show high levels of EGFP and RFP expression. Co-transfection of *PRE*–*EGFP *+ *pActin*-*RFP* reporters with *Pc* **b** or *E(z)* **c** over-expressing constructs resulted in strong repression of EGFP but had no effect on RFP. Over-expression of a non-PcG protein, *DNApol*-*α50* **d** shows no effect on *PRE*-*EGFP* as both EGFP and RFP expression levels are comparable to those of **a**. Merge images show comparable RFP signal and cell density. **e** Total cell lysates from transfected cells **a**–**d** were probed with anti-FLAG, anti-GFP and anti-tubulin antibodies on a Western blot. Over-expression of both PC and E(z) shows a drastic reduction in EGFP expression while DNApol-α50 did not significantly change EGFP levels. **f** Over-expression of PC represses *PRE*-*F.Luc* in a dose-dependent manner. Increased repression of *F.Luc* was observed with increasing amounts (1, 2, 4 ng) of transiently transfected *Pc* over-expression construct, *pActin*-*Pc*. **g** Knockdown of known members of *trxG* (*trx*, *ash1*, *brm*, *mor*) leads to decrease in F.Luc expression in cells transiently transfected with *PRE*-*F.Luc* along with *pActin*-*R.Luc* (*Renilla* Luciferase) used as a normalization control. Knockdown of *F.Luc* revealed strong repression of reporter. dsRNA against *LacZ* was used as a negative control. Relative F.Luc values, normalized against R.Luc, used as an internal control, recorded at 72 and 96 h after transfection are shown. All knockdowns of trxG genes resulted in significant downregulation of relative F.Luc levels (*p* < 0.0001) at both time points. Experiments shown in **f** and **g** were performed in triplicates in two different sets and were analyzed by student *t* test **f** or one-way ANOVA **g** (*p ≤ 0.05, **p ≤ 0.01, ***p ≤ 0.001 or ****p ≤ 0.0001). Error bars represent SEM

Although several *PRE*-based transgenes have been shown to maintain stable and heritable gene expression of reporter genes in *Drosophila* [19], their analyses involve time-consuming dissections and staining that cannot be easily optimized for a genome-wide RNAi screen. We aimed at developing a robust cell-based reporter assay in *Drosophila*, sensitive to the levels of PcG/trxG, that could help us discover novel regulators of trxG. To this end, we adapted the *PBX*-*bxd*-*IDE*-*LacZ* reporter that has been previously characterized in flies [23]. In this reporter, bacterial *LacZ* gene is regulated by *Drosophila Ubx* (*Ultrabithorax*) promoter [24] and *bxd-PRE* [25] along with *PBX* (*postbithorax*) and *IDE* (*Imaginal Disc Enhancer*) enhancers of *Ubx* [26]. We modified *PBX*-*bxd*-*IDE*-*LacZ* by replacing *LacZ* with *Enhanced Green Fluorescent Protein* (*EGFP*) (hereafter referred to as *PRE*-*EGFP*) (Additional file 1: Fig. S1a) to monitor EGFP expression in fly cells. We hypothesized that in *Drosophila* cells transfected with the *PRE*-*EGFP* reporter, depletion of *trxG* or over-expression of *PcG* genes would result in a decreased EGFP expression (Additional file 1: Fig. S1b). To validate the sensitivity of our reporter, *PRE*-*EGFP* was transiently transfected in *Drosophila* cells alone (Fig. 1a) or with constructs over-expressing either *Pc* (*Polycomb*) (Fig. 1b) or *E(z)* (*Enhancer of Zeste*) (Fig. 1c). To rule out any variations in transfection efficiency, *Actin* promoter-driven RFP (*pActin*-*RFP*) was also co-transfected with *PRE*-*EGFP* (Fig. 1a–d). As compared to cells transfected with *PRE*-*EGFP* alone (Fig. 1a), there was a marked decrease in the number of EGFP positive cells when either *Pc* (Fig. 1b) or *E(z)* (Fig. 1c) were over-expressed while the number of RFP-positive cells remained constant. To evaluate the specificity of the reporter, *DNApol*-*α50*, a nuclear factor not involved in PcG-mediated repression, was co-transfected with *PRE*-*EGFP* (Fig. 1d). Over-expression of *DNApol*-*α50* had no effect on *PRE*-*EGFP*. This was further confirmed by Western blot of total cell lysates from the transfected cells with anti-GFP antibody (Fig. 1e). To monitor the impact of perturbations in trxG more quantitatively and to develop a robust reporter for high-throughput RNAi screens, *Firefly Luciferase* (*F.Luc*) gene was cloned instead of *EGFP* in the *PRE*-*EGFP* construct (hereafter referred to as *PRE*-*F.Luc*) (Additional file 1: Fig. S1a). *Drosophila* cells transfected with *PRE*-*F.Luc* together with increasing amounts of *Pc* over-expression construct showed a dose-dependent decrease in the amount of relative F.Luc activity (Fig. 1f). To further validate that *PRE*-*F.Luc* could be used to discover factors that impact trxG-mediated gene regulation, cells transfected with *PRE*-*F.Luc* were subjected to knockdown of known trxG members and the resultant F.Luc activity was monitored (Fig. 1g). Treatment of cells with dsRNAs against *trithorax* (*trx*), *absent small or homeotic discs1* (*ash1*), *brahma* (*brm*) and *moira* (*mor*) resulted in a significant decrease in the amount of F.Luc expression as compared to cells treated with dsRNA against *LacZ*, indicating the specific silencing of *PRE*-*F.Luc* reporter. The sensitivity of *PRE*-*F.Luc* reporter to the changing levels of PcG and trxG corroborated with our hypothesis and allowed us to use *PRE*-*F.Luc* in a high-throughput cell-based RNAi screen to identify novel players in trxG-mediated gene regulation.

### Genome-wide RNAi screen reveals novel trxG-like factors

**Fig. 2:**
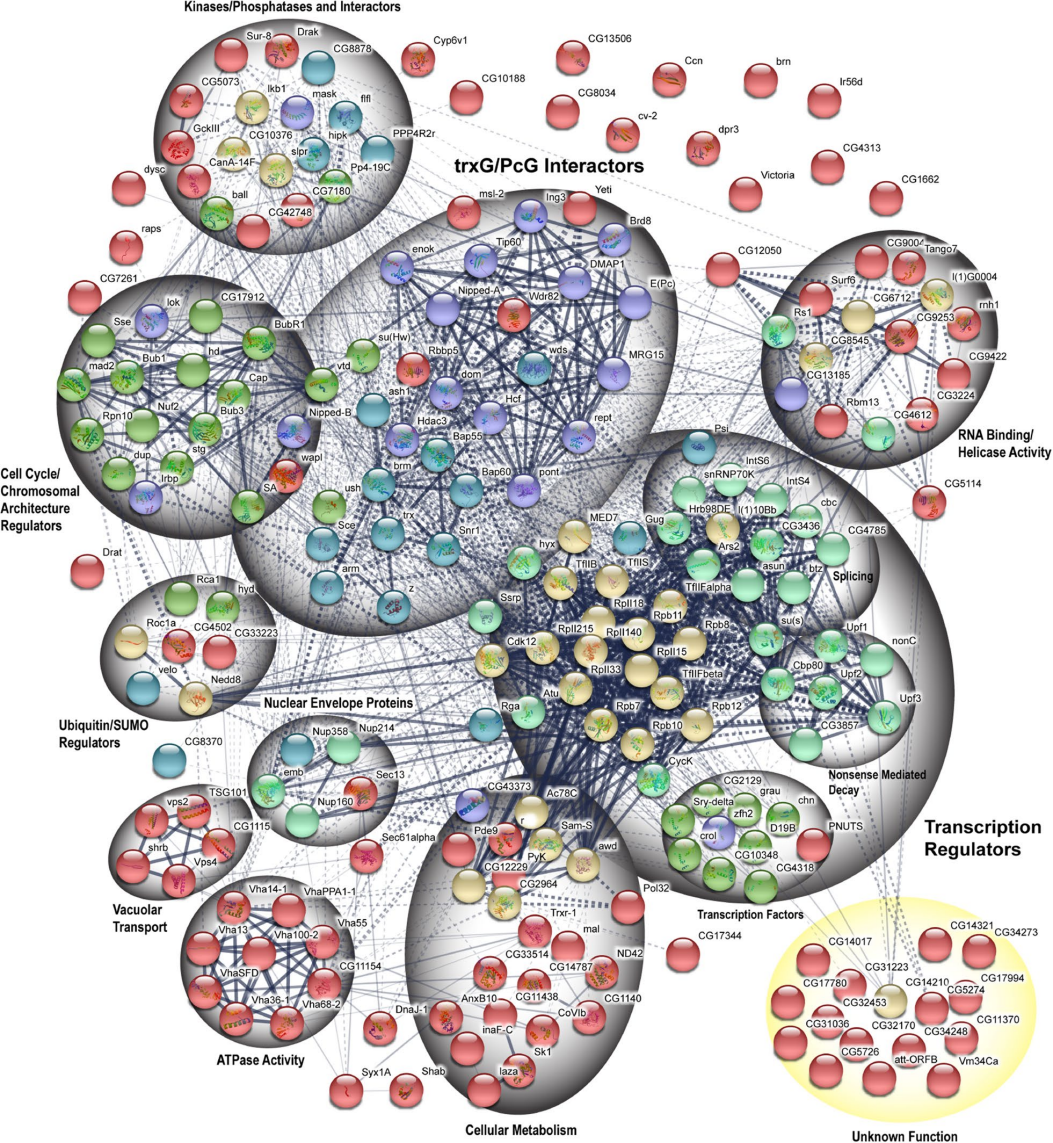
Protein interaction map of the candidates identified in the screen was generated using STRING database [61]. Proteins (nodes) in the network are connected by edges of varying thickness (depicting degree of confidence). Functionally related proteins were clustered together (grey circles) whereas proteins with unknown functions were marked separately (yellow circle)

A genome-wide RNAi screen was carried out using dsRNAs from *Drosophila* Heidelberg 2 (HD2) library, covering about 98% of the annotated *Drosophila* genome [27, 28]. Importantly, dsRNAs against known trxG members (*trx, ash1*) and specific reporter gene (*F.Luc*) were used as positive controls, whereas dsRNA against *LacZ* was used as a negative control in all plates. Along with *PRE*-*F.Luc* reporter construct, *Actin* promoter-driven Renilla Luciferase (*pActin*-*R.Luc*) was co-transfected as a normalization control to exclude possible artifacts such as cell death and effect on general transcription. The experimental strategy followed for the screen is summarized in Additional file 2: Fig. S2a (for details see “Materials and methods”). Based on the Z scores obtained from positive controls (*trx, ash1*), cut-offs were defined for the screen (Additional file 2: Fig. S2b, c) and a list of 217 potential trxG candidates was generated (Additional file 3: Table S1). Importantly, several known members of trxG appeared in the list of candidate genes, thus validating the efficiency of our assay. A high-resolution interaction map of the candidate trxG genes identified in our screen highlights the presence of trxG interacting partners including MRG15 [29], Pontin [30, 31], smc3 [32] and wapl [33] (Fig. 2). Further analysis revealed our candidate genes to be involved in a multitude of cellular processes including cell division, cell fate determination, development and cell signaling. From our top scoring candidates, we selected *enoki mushroom* (*enok*) for detailed genetic and molecular analyses because of its known interaction with PC [34], Ash1 [35] and involvement in gene activation during oogenesis in flies [36].

### Mutants of *enok* behave like *trxG* mutants

**Fig. 3:**
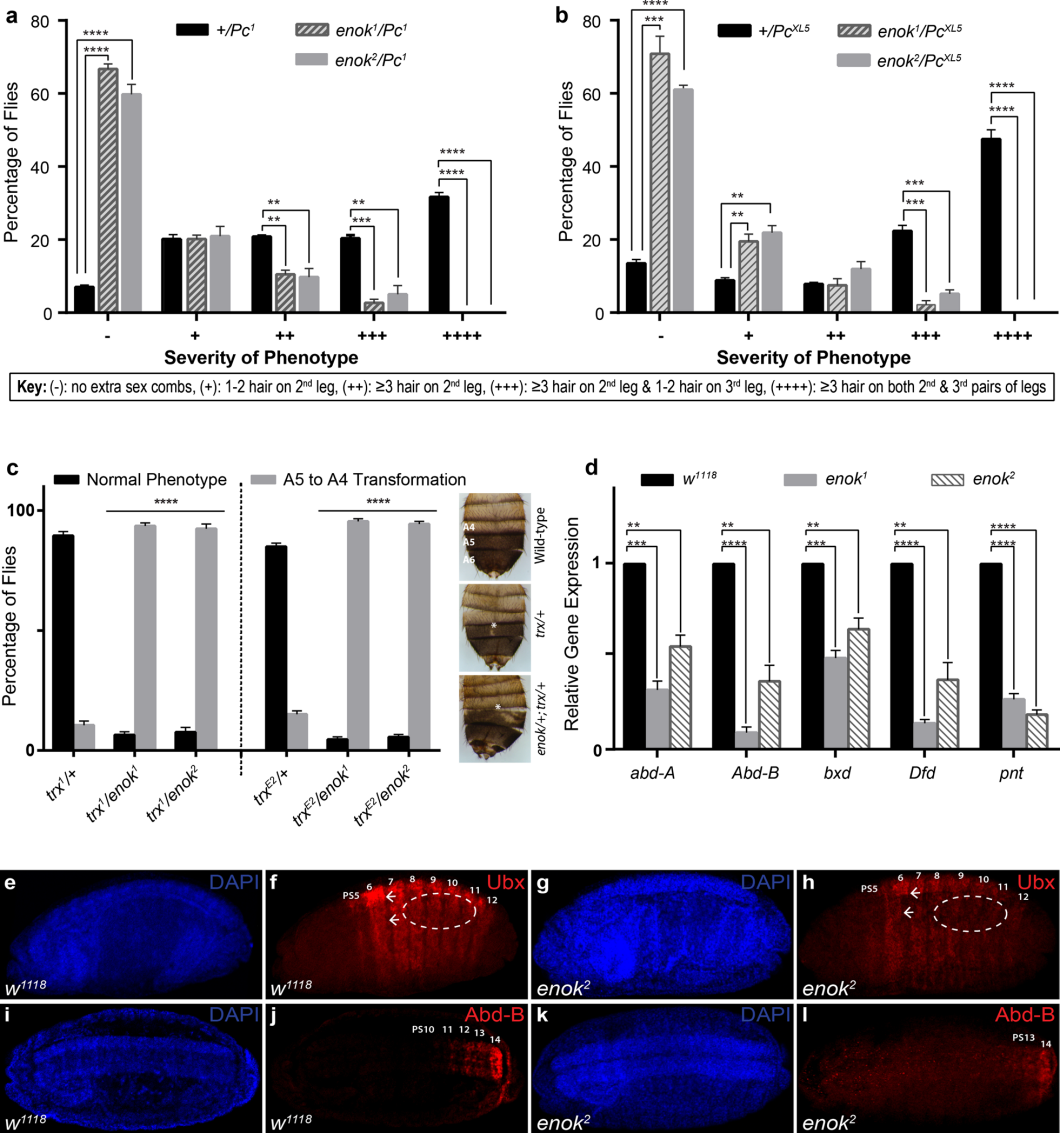
Mutants of *enok* genetically interact with *Pc* and *trx.* Two mutants of *enok* (*enok*^*1*^ and *enok*^*2*^) crossed to either *Pc*^*1*^ (**a**) or *Pc*^*XL5*^ (**b**) alleles strongly suppressed extra sex comb phenotype. +*/Pc* heterozygotes from *Pc* alleles (*Pc*^*1*^ and *Pc*^*XL5*^) crossed to *w*^*1118*^ flies represent control and showed strong extra sex combs. More than 150 male flies with the desired genotype (*enok* mutant/*Pc* mutant) in the progeny of each cross were analyzed. Based on the number of extra sex comb hairs on 2nd and 3rd legs, male flies were categorized (see key) [62]. **c** *enok*^*1*^ and *enok*^*2*^ were crossed to *trx*^*1*^ and *trx*^*E2*^ alleles and males in the progeny were scored for loss of pigmentation (A5 to A4 transformation, white asterisk). *trx/*+ heterozygotes from *w*^*1118*^ crossed to *trx* mutants served as control. As compared to *trx*/+ , double mutants (*enok*; *trx*) showed strong enhancement of *trx* mutant phenotype (A5 to A4 transformation). **a**–**c** Percentage of flies in each category was plotted as bar graphs with SEM. **d** Effect of *enok* mutations on the expression of homeotic (*abd*-*A, Abd*-*B, Ubx*, *Dfd)* and non-homeotic (*pnt*) genes. qRT-PCR performed on 20-h-old homozygous *enok*^*1*^ or *enok*^*2*^ embryos compared to *w*^*1118*^ embryos of the same age. A drastic reduction was observed in the expression of all the genes analyzed. **e**–**h** As compared to Ubx expression pattern (PS5-PS12) in *w*^*1118*^ used as a control (**e, f**), a strongly reduced Ubx expression is seen in stage 15 homozygous *enok* embryos. Misaligned (arrows) and merged (oval) parasegmental boundaries of Ubx staining patterns are visible (**g, h**). As compared to *w*^*1118*^ embryos (**i, j**), immunostaining of *enok* mutant embryos showed strongly reduced Abd-B expression restricted to PS13-14 only. **a**–**d** Experiments were performed in triplicates and individual Student’s *t* tests were performed for each category and the *p* values obtained were marked as **p ≤ 0.01, ***p ≤ 0.001 or ****p ≤ 0.0001. Error bars represent SEM

Although *Drosophila* Enok interacts with PcG [34, 35] and is involved in acetylation of histone H3K23 [36], the physiological relevance of Enok in epigenetic cellular memory remained elusive. To investigate whether *enok* genetically interacts with the PcG/trxG system, two mutant alleles of *enok* were crossed to two different alleles of *Pc* (*1*, *XL5*). *Pc* heterozygous mutants show a strong extra sex comb phenotype. Importantly, both mutant alleles of *enok* strongly suppressed the extra sex comb phenotype (Fig. 3a, b). Since *enok* mutants strongly suppressed extra sex comb phenotype, we examined the genetic interaction of *enok* with *trx* by crossing *enok* mutant flies with two different alleles of *trx* (*1*, *E2*). As compared to wild type, *trx* heterozygous mutants (*trx*^*1*^*/*+ or *trx*^*E2*^*/*+) show A5 to A4 transformation (Fig. 3c) indicated by loss of pigmentation in A5 [37]. As compared to *trx* heterozygotes, a strong A5 to A4 transformation was observed in *enok/*+ ; *trx/*+ double-mutant flies (Fig. 3c). Both the alleles of *enok* strongly enhanced A5 to A4 transformations when crossed with either *trx*^*1*^ or *trx*^*E2*^. These genetic analyses suggest that *enok*, like classic trxG genes, strongly suppresses homeotic transformations caused by *Pc* and enhances those caused by *trx* mutations.

To characterize *enok* at the molecular level, we questioned if mutations in *enok* alter gene expression patterns of homeotic and non-homeotic targets of trxG in fly embryos because homozygous *enok* mutants do not survive to adulthood. As compared to *w*^*1118*^ embryos, *enok*^*1*^ and *enok*^*2*^ homozygous embryos showed a drastic reduction in expression of *abdominal*-*A* (*abd*-*A*), *Abdominal*-*B* (*Abd*-*B*), *Ultrabithorax* (*Ubx*), *Deformed* (*Dfd*) and *pointed* (*pnt*) in real-time PCR analysis (Fig. 3d). Additionally, stage 15 homozygous *enok* mutant embryos showed aberrant patterns of Ubx and Abd-B staining as compared to *w*^*1118*^ embryos (Fig. 3e–l). At this stage, Ubx expression is weak in parasegment 5 (PS5), highest in PS6 and progressively decreases from PS7-12 (Fig. 3e, f). However, homozygous *enok* mutant embryos displayed strongly diminished Ubx expression in all these regions (Fig. 3g, h), similar to *trx* mutations [38, 39]. Moreover, stripes of Ubx expression appear misaligned and merged across different parasegments (Fig. 3g, h). Normal expression of Abd-B progressively increases from PS10 to PS14 (Fig. 3i, j), whereas *enok* mutant embryos displayed loss of expression of Abd-B in PS10–PS12, resulting in a shift of Abd-B expression boundary to PS13–PS14 only (Fig. 3k, l). The decreased expression of Abd-B correlates with the increased A5 to A4 transformation observed in Fig. 3c and is also known to be associated with trxG mutations [40, 41]. These results indicate that *enok* interacts with trxG at the genetic level and is involved in the regulation of trxG target genes.

### Enok maintains active state of trxG targets by inhibiting PC

**Fig. 4:**
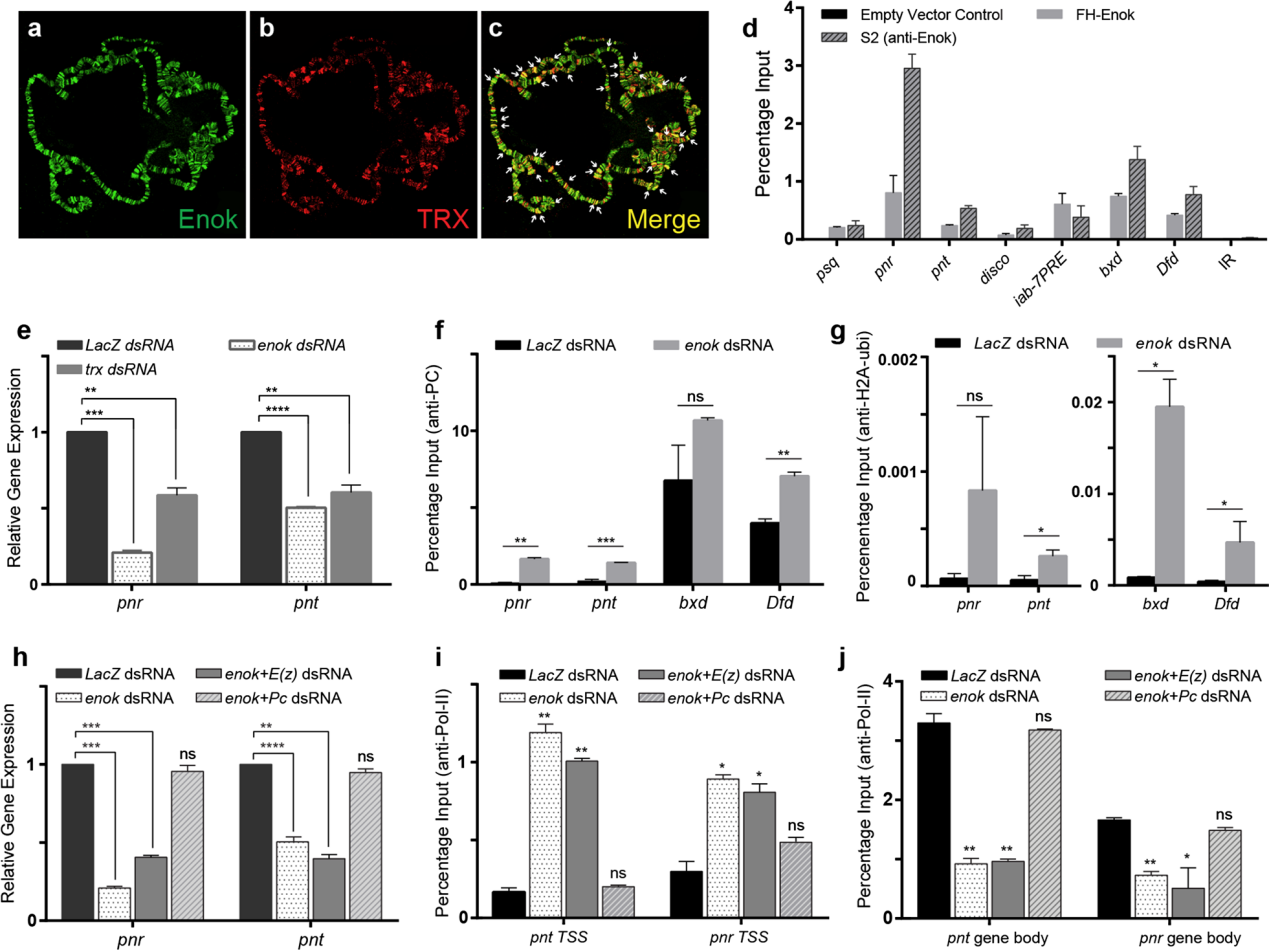
Enok associates with chromatin and facilitates trxG by inhibiting PC binding. **a**–**c** Polytene chromosomes from third instar larvae expressing Myc-tagged-Enok were stained with anti-Myc (**a**) and anti-TRX (**b**) antibodies. Enok and TRX colocalize at several loci (**c**, arrows). **d** ChIP with anti-FLAG antibody using stable cells expressing FLAG-tagged-Enok (FH-Enok) showed enrichment of Enok at non-homeotic (*psq, pnr*, *pnt*, *disco*) and homeotic (*iab*-*7, bxd*, *Dfd*) targets when compared with anti-FLAG ChIP from empty vector control cell line. Enok was absent on an Intergenic Region (IR) used as negative control. ChIP from S2 cells using anti-Enok antibody showed strong enrichment of Enok at trxG targets mentioned above. **e** Effect of *enok* knockdown on *pnr* and *pnt* in D.Mel-2 cell line. Cells treated with dsRNA against *enok* showed a trend similar to that of *trx* knockdown, with decreased expression of *pnr* and *pnt* when compared with *LacZ* dsRNA-treated cells (control). **f** ChIP with anti-PC using *enok-*depleted cells showed increased enrichment of PC at *pnr*, *pnt*, *bxd* and *Dfd* when compared to *LacZ* dsRNA-treated control cells. **g** ChIP with anti-H2A-ubi from cells treated with dsRNA against *enok* showed increased enrichment of H2AK118ub1 on *pnr*, *pnt, bxd* and *Dfd* as compared to control cells. **h** Cells exposed to dsRNA against both *enok* and *E(z)* showed a decrease in transcript levels of *pnr* and *pnt* in a manner similar to *enok* knockdown. Cells treated with dsRNA against both *Pc* and *enok* showed *pnr* and *pnt* expression levels comparable to control cells. **i**, **j** ChIP with Pol-II antibody from cells treated with dsRNA against *enok* alone, *enok *+ *E(z)* or *enok *+ *Pc*. Knockdown of *enok* alone or *enok *+ *E(z)* showed increased occupancy of Pol-II at TSS (**i**) and decreased occupancy in gene body (**j**) of *pnt* and *pnr*. In contrast, *enok *+ *Pc* knockdown revealed Pol-II levels similar to control cells. Knockdown experiments shown in (**e**, **h**) were performed in triplicates and ChIP experiments shown in (**d**, **f**, **g**, **i** and **j**) were performed in duplicates. Individual Student’s *t* tests were performed and the *p* values obtained were marked as ns: not significant, *p ≤ 0.05, **p ≤ 0.01, ***p ≤ 0.001 or ****p ≤ 0.0001. Error bars represent SEM

To investigate a potential molecular link between Enok and trxG, we generated a *Drosophila* transgene expressing *enok* coding sequence fused with Myc tag, under *UAS* promoter (Additional file 4: Fig. S3a). Immunostaining of polytene chromosomes from third instar larvae of *UAS*-*enok*-*Myc* crossed with *sgs*-*GAL4* revealed association of Enok with polytene chromosomes primarily at the interband regions (Fig. 4a–c). Importantly, there was a significant overlap between TRX and Enok-binding sites. In addition, a significant overlap between PC and Enok was also observed on polytene chromosomes (Additional file 4: Fig. S3b–e).

Chromatin-binding profile of Enok on TRX-binding sites was validated by ChIP analysis of chromatin from cells expressing FLAG-tagged Enok (Additional file 4: Fig. S3f). FLAG-Enok was observed at the transcription start sites (TSS) of *pipsqueak* (*psq*), *pannier* (*pnr*), *pointed* (*pnt*) and *disconnected* (*disco*) which are known binding sites of PcG/trxG [42–45]. Moreover, Enok was also found to be associated with *iab*-*7PRE*, *bxd* and *Dfd* regulatory regions of homeotic genes (Fig. 4d), known to be bound by both PC and TRX proteins [46]. In contrast, ChIP from empty vector control cells resulted in negligible enrichment at all the regions analyzed. Enok binding on TRX-binding sites was further validated by ChIP using anti-Enok antibody on wild-type *Drosophila* S2 cells. A similar pattern, albeit with a stronger enrichment, was seen on all the targets analyzed (Fig. 4d). Importantly, Enok was absent on an intergenic region that is not bound by TRX [47].

Next, we questioned how Enok facilitates trxG-mediated gene activation. Similar to the effect of *trx* knockdown, depletion of *enok* in *Drosophila* cells (Additional file 4: Fig. S3 g) resulted in a significant decrease in mRNA levels of *pnr* and *pnt* (Fig. 4e) that are known non-homeotic targets of trxG [16]. Knockdown of *enok*, however, did not significantly alter the amount of *trx* mRNA in the cells (Additional file 4: Fig. S3h). Depletion of *enok* also did not cause a change in the enrichment of TRX protein (Additional file 4: Fig. S3i) or trxG-associated histone modification, H3K27ac (Additional file 4: Fig. S3j), on trxG target genes when analyzed by ChIP. We, therefore, hypothesized that Enok might be essential for trxG-mediated gene activation not by enhancing trxG function but by specifically antagonizing the activity of PcG. Hence, we analyzed the association of PC at active (*pnr, pnt*) and silent (*bxd, Dfd*) targets after the knockdown of *enok* in cells (Fig. 4f). Depletion of *enok*, resulting in diminished H3K23ac (Additional file 4: Fig. S3k), showed a greater enrichment of PC at all sites analyzed by ChIP (Fig. 4f). Moreover, enhanced levels of PRC1-associated H2AK118ub1 were also found at active and silent loci in cells with *enok* knockdown (Fig. 4g). Interestingly, in *enok*-depleted cells, there was no difference in the enrichment of E(z) and H3K27me3 levels when compared to *LacZ* dsRNA-treated cells (Additional file 4: Fig. S3 l, m). Together, these results highlight a PRC1-specific role of Enok in counteracting repression to maintain active gene expression.

To further investigate the role of Enok in antagonizing PRC1-mediated repression, cells were treated with either *enok* dsRNA alone or in combination with dsRNA against *Pc* (*enok *+ *Pc*) or *E(z)* (*enok *+ *E(z)*) (Fig. 4h–j). Interestingly, gene expression levels of *pnr* and *pnt* were significantly reduced in *enok* and *enok *+ *E(z)* depleted cells when compared to *LacZ* dsRNA-treated cells. In contrast, *enok *+ *Pc* knockdown cells showed *pnr* and *pnt* expression levels comparable to the *LacZ* dsRNA-treated control (Fig. 4h). Since depletion of *enok* and *Pc* together resulted in near wild-type levels of gene expression, we concluded that Enok works specifically to counteract PRC1 and contributes to anti-silencing act of trxG.

Next, we determined the occupancy of RNA Polymerase II (Pol-II) at *pnt* and *pnr*. We found an increased enrichment of Pol-II at the TSS of *pnt* and *pnr* in *enok* and *enok *+ *E(z)*-depleted cells (Fig. 4i), when compared to *LacZ* dsRNA-treated cells. This increase in Pol-II occupancy at the TSS was accompanied by decreased Pol-II enrichment in the gene body of *pnt* and *pnr* (Fig. 4j). These findings, along with the decreased expression of *pnt* and *pnr* (Fig. 4h), indicate stalling of Pol-II [48, 49] at the TSS of these trxG targets. In contrast, as compared to cells with knockdown against *enok* alone or *enok *+ *E(z)*, cells treated with dsRNA against *enok *+ *Pc* showed a decreased enrichment of Pol-II at the TSS (Fig. 4i) and an increased enrichment in the gene body (Fig. 4j). This result, along with the restoration of expression levels of *pnt* and *pnr* in *enok *+ *Pc* (Fig. 4h)-depleted cells, indicated a release of Pol-II from the paused state. Taken together, our results suggest that depletion of *enok* leads to repression of trxG targets due to increased PRC1 recruitment which prevents Pol-II from transcribing its target genes.

## Discussion

We have developed an ex vivo approach that led to the discovery of several new genes regulating trxG-mediated gene activation. Using a well-characterized *bxd-PRE*-reporter [23], comprising of *Ubx* promoter and enhancers, we developed a cell-based assay and performed a genome-wide RNAi screen in *Drosophila*. Based on the Z scores of *trx* and *ash1* knockdown, we defined a stringent cut-off and identified more than 200 genes affecting the reporter in a manner similar to trxG genes. Identification of known members of trxG and their interactors as well as chromatin modifiers in the genome-wide RNAi screen validated our reverse genetics approach and efficacy of the reporter system to discover new regulators of trxG. Moreover, presence of chromatin modifiers like members of TIP60 complex and proteins associated with RNA polymerase II, known to interact with trxG, further substantiates that regulators of gene activation were predominantly identified. Although we identified only a subset of known trxG members in our screen, failure to identify all can be attributed to the highly context-dependent working of PcG/trxG system [50]. Since two specific enhancers of Ubx drive the expression of our reporter, it might be regulated by only a subset of trxG members, which could further explain the failure to identify all members of trxG. Interestingly, some of the top scoring candidates in our screen were also recently found to be a part of the interaction network of GAGA factor, a known trxG member [51].

We characterized trxG-like behavior of Enok, and established its genetic and molecular link with trxG. Although *Drosophila* Enok has previously been shown to interact with PC [34] and Ash1 [35], its physiological relevance with PcG/trxG or epigenetic cellular memory remained elusive. Our results demonstrate that *enok* behaves like a trxG gene, by antagonizing PcG, and is essential for maintaining active gene expression in *Drosophila*. Appearance of extra sex combs in *Pc* heterozygous males is a consequence of ectopic activation of homeotic genes which relies upon the trxG. However, depletion of trxG proteins counteracts the reduced dose of PC, restoring normal regulation of homeotic genes and suppressing the extra sex comb phenotype [8]. Strong suppression of extra sex comb phenotype by two different mutants of *enok* illustrates that it acts as a trxG gene, consequently counteracting repression maintained by PcG. This finding is further supported by the fact that both mutant alleles of *enok* strongly enhance *trx* mutant phenotype, which also corroborates with drastic reduction in transcript levels of trxG target genes in embryos lacking functional *enok*. A significant overlap between Enok and TRX at chromatin further validates our genetic analysis. Since depletion of *enok* led to increased PC binding and enhanced H2AK118ub1 at trxG targets, we suggest that Enok may specifically inhibit PRC1 and facilitate anti-silencing activity of trxG. In contrast, no change in enrichment of E(z) and its associated mark, H3K27me3, was observed at TSS of trxG targets in cells with reduced *enok*, indicating recruitment of PRC1 in a potentially H3K27me3-independent manner. Such PRC2-independent recruitment of PRC1 has also been reported previously [52].

**Fig. 5:**
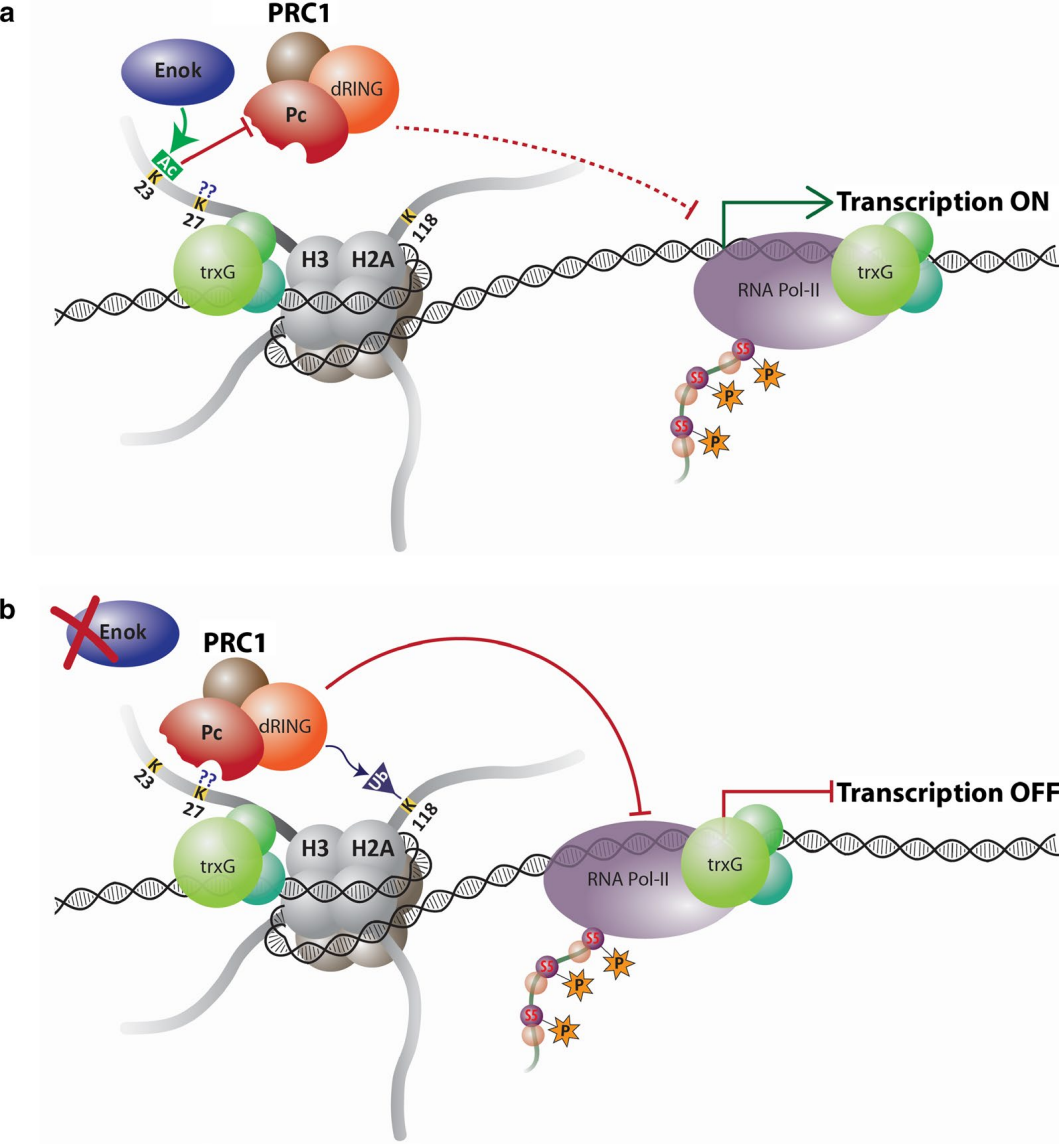
Proposed model depicting antagonistic effect of Enok on PRC1. **a** At active trxG target genes, Enok acetylates H3K23 which inhibits PRC1 binding and consequent transcriptional block of RNA-Pol-II. As a consequence, genes remain in an active state. **b** In the absence of Enok, H3K23 remains unmodified which allows PRC1 recruitment to the chromatin and addition of H2AK118 mono-ubiquitination. Subsequently, PRC1 holds RNA-Pol-II in a stalled state, leading to gene silencing

In light of our results, we propose that Enok counteracts PRC1-mediated block of transcription, evident in the form of stalled Pol-II at the TSS of *pnr* and *pnt* in cells with depleted *enok*. Molecular interaction of Enok with PRC1 on developmental genes in flies and humans [35] further supports the notion that Enok facilitates trxG by inhibiting PRC1. In mice, MOZ (homolog of Enok) is known to play an antagonistic role to PcG member BMI1 in regulating Hox genes [53]. In agreement with the finding that PC chromodomain binding to H3K27me3 requires an unmodified H3K23 [54], our data suggest that Enok-mediated H3K23ac inhibits binding of PC to its target genes. We propose that in the presence of Enok at active loci, acetylated H3K23 inhibits binding of PRC1 leading to increased transcriptional activity of Pol-II (Fig. 5a). On the other hand, loss of Enok leads to decreased H3K23ac, thus allowing PRC1 binding and consequent stalling of Pol-II at TSS (Fig. 5b). Since Enok was also found to associate with silent loci (*bxd*, *Dfd*, *iab*-*7*) and interact with PRC1, we suggest that Enok is kept in an inactive state on these loci by PC in a manner similar to the inhibitory interaction between PC and CBP [55]. Further molecular and biochemical characterization of this intricate relationship between PcG and Enok will help discover how trxG maintains dynamic gene expression patterns during development.

## Conclusion

In summary, we have developed a cell-based assay for an ex vivo genome-wide RNAi screen to identify potential trxG regulators in *Drosophila*. Our RNAi screen led to the discovery of more than 200 genes which perturbed our luciferase-based reporter in a manner similar to known trxG members. We have also provided evidence that Enok, a top trxG candidate in our screen, contributes to anti-silencing action of trxG by counteracting PcG proteins. We propose that H3K23 acetylation by Enok counteracts PcG-mediated suppression by inhibiting PRC1 recruitment, contributing to gene activation. Genetic and molecular evidence obtained suggests that Enok interacts with trxG and as a result with their major developmental regulatory targets, thus providing a possible molecular link through which it could influence epigenetic cell memory.

## Materials and methods

### Antibodies

Following antibodies were used in this study: mouse α-GFP (Roche, 11814460001, WB:1:5000), mouse α-Tubulin (Abcam, ab44928, WB: 1:2000), mouse α-Ubx (DSHB, Fp3.38, IF: 1:20), mouse α-Abd-B (DSHB, 1A2E9, IF: 1:40), rabbit α-TRX (gift from R. Paro, IF: 1:20, ChIP: 5 µl), rabbit α-PC (Santa Cruz, D220, IF: 1:20, ChIP: 2 µl), rabbit α-E(z) (gift from R. S. Jones, ChIP: 2 µl), rabbit α-H3K27ac (Abcam, ab4729, ChIP: 2 µl), mouse α-H3K27me3 (Abcam, ab6002, ChIP: 2 µl), mouse α-Myc (Santa Cruz, 9E10, WB: 1:1000, IF: 1:50), mouse α-FLAG M2 (Sigma Aldrich, WB: 1:2000, ChIP: 5 µl), rabbit α-Enok (gift from J. Workman, WB: 1:1000, ChIP: 5 µl), mouse α-H2A-ubi (Millipore, 05-678, ChIP: 5 µl), rabbit α-H3K23ac (Millipore, 07-355, WB: 1:10,000) and mouse α-RNA polymerase II (Abcam, ab5408, ChIP: 1 µl).

### *Drosophila* cell culture

*Drosophila* S2 cells were cultured in Schneider’s *Drosophila* medium (Gibco, ThermoFisher Scientific), supplemented with 10% fetal bovine serum (Gibco, ThermoFisher Scientific) and 1% penicillin–streptomycin (Gibco, ThermoFisher Scientific) at 25 °C. Schneider S2 cells adjusted to serum-free growth medium (D.Mel-2, Invitrogen) were cultured in Express Five SFM (Gibco, ThermoFisher Scientific) supplemented with 20 mM GlutaMAX (Gibco, ThermoFisher Scientific) and 1% penicillin–streptomycin.

### Construction of reporter plasmid

The Carnegie 20 fly transformation vector, *PBX*-*PRE1.6*-*IDE*-*Ubx promoter*-*LacZ* was a kind gift from Jürg Müller [23]. The plasmid was modified to replace *LacZ* with *EGFP* or *F.Luc* (*Firefly* Luciferase) using *Sal* I restriction sites. Since the ~ 24 kb plasmid was not suitable for efficient transfection in cell culture, the cassette containing *PBX*-*PRE1.6*-*IDE*-*Ubx promoter* was cloned into a smaller cell culture vector *pCoBlast* (ThermoFisher Scientific) using the following strategy: a *Sal* I restriction site was added downstream of *Not* I in the Multiple Cloning Sites (MCS) of *pCoBlast* using linkers. The *PBX*-*PRE1.6*-*IDE*-*Ubx promoter* was excised from the fly transformation vector by digesting with *Not* I and *Sal* I and cloned into the modified *pCoBlast* at *Not* I and *Sal* I sites. The reporter genes *EGFP* and *F.Luc* were then PCR amplified from the respective fly vectors, along with the *Hsp70*-*PolyA* tail, using primers with an *Xho* I restriction site which were cloned at *Sal* I in the *pCoBlast*-*PBX*-*PRE1.6*-*IDE*-*Ubx* vector. *pActin*-*RFP* and *pActin*-*R.Luc* (*Renilla* Luciferase) were used as internal control reporter plasmids. Primer details can be found in Additional file 5: Table S2.

### *PRE*-*EGFP* reporter assay

S2 cells (1x10^6^) were transfected with *PRE*-*EGFP* reporter alone (0.5 µg) or together with constructs expressing full-length *Pc* [*pMT*-*FLAG*-*His*-*Pc*] (0.5 µg), full-length *E(z)* [*pMT*-*FLAG*-*His*-*E(z)*] (0.5 µg) or full-length *DNApol*-*α50* [*pMT*-*FLAG*-*His*-*DNApol*-*α50*] (0.5 µg) using Effectene reagent as per recommended protocol (Qiagen). Expression of *Pc*, *E(z)* and *DNApol*-*α50* was induced with 500 µM CuSO_4_. After 72 h of induction, EGFP and RFP signals were monitored using a confocal microscope (Nikon C2). Total cell lysates were analyzed by Western blot.

### *PRE*-*F.Luc* reporter assay

dsRNA was introduced into cells by the bathing protocol described previously [56]. 250 ng dsRNA was preloaded into 384-well plates (Greiner). Each plate had positive and negative controls (see results). Subsequently, 10,000 (~ 30 μl) D.Mel-2 cells were dispensed per well using a MultiDrop (ThermoLab systems). After 24-h incubation at 25 °C, the cells were transfected with 0.005 μg of a DNA mix (0.002 µg of *PRE*-*F.Luc*, and 0.003 µg of *pActin*-*R.Luc*) per well using Effectene reagent. 3 days post-transfection, cells were lysed to measure F.Luc and R.Luc (*Renilla* Luciferase) activities using a dual luminescence assay on a Mithras LB940 plate reader (Berthold Technologies). Each experiment was performed in triplicates within two different replicas.

For the over-expression assay of *Pc*, 0.005 μg of a DNA mix (0.002 µg of *PRE*-*F.Luc* and 0.003 µg of *pActin*-*R.Luc*) was transfected alone or with increasing concentrations (0.001, 0.002 and 0.003 µg) of *pActin*-*FLAG*-*Myc*-*Pc*.

### dsRNA synthesis

Templates for the preparation of dsRNAs were amplified by PCR from cDNA or genomic DNA using T7-tailed oligonucleotides as primers. These templates were then used for in vitro transcription to synthesize dsRNA using T7 Megascript kit (Ambion) following the manufacturer’s instructions. Primers for all the known members of trxG were chosen from second-generation *Drosophila* dsRNA library (Heidelberg 2) [27]. Complete primer and amplicon sequence information can be found at http://rnai.dkfz.de.

### Genome-wide RNAi screen

dsRNA was synthesized using the second-generation *Drosophila* dsRNA library (Heidelberg 2) as described [57]. Bathing of D.Mel-2 cells with dsRNA against whole genome [57] was carried out as described above. Each gene in the HD2 library was knocked down in triplicates and the entire experiment was carried out in duplicates. Each plate contained controls (see “Results”) in triplicates. 5 days from the start of the experiment, F.Luc and R.Luc values were recorded using a dual luminescence reader as described [58]. The ratio of the experimental reporter F.Luc to the invariant co-reporter R.Luc values was calculated to exclude possible artifacts, such as cell death and effect on general transcription. Knockdown of genes that affected both F.Luc and R.Luc was removed from further analysis. Relative F.Luc and R.Luc expressions were averaged for both replicates. Results were exported into excel sheets and were analyzed using the guidelines described previously [59]. Instead of using 99.99% confidence values as cut-offs, more stringent cut-offs were defined based on *trx* and *ash1* Z scores (higher Z score). A number of ribosomal proteins, translational initiation or elongation factors were also found affecting *PRE*-*F.Luc*. Since these genes are known to appear in multiple genome-wide RNAi screens as an unexplained group of hits [60], all such factors were excluded from the list of candidates (Additional file 3: Table S1).

### Protein interaction analysis

An interaction map of candidates identified as potential trxG regulators in the genome-wide RNAi screen was made using the STRING database [61]. A custom confidence value of 0.300 was used. The network was grouped into six clusters using k-means. Since many proteins were involved in multiple processes, the clusters were then manually modified to refine the functional grouping of the proteins.

### Western blotting

Cells were lysed in cold lysis buffer (150 mM NaCl, 0.05 M Tris, 1% Triton X-100) supplemented with protease inhibitors, supernatants were collected in fresh tubes and mixed with 2X reducing sample buffer and boiled at 95 °C for 5 min. The proteins were resolved on 12% SDS-PAGE and transferred onto nitrocellulose membranes that were blocked with 5% milk for 2 h before probing with the appropriate antibodies overnight at 4 °C. Secondary antibodies, HRP conjugated, were used at 1:10,000 dilution and blots were developed using ECL reagents (GE Healthcare).

### Fly strains

The following fly strains were obtained from Bloomington *Drosophila* Stock Center: *enok*^*1*^ (BN 6284), *enok*^*2*^ (BN 6285), *Pc*^*XL5*^*/TM3Ser,Sb*, *Pc*^*1*^*/TM3Ser*, *trx*^*1*^ (BN 2114), *trx*^*E2*^ (BN 24160).

The following strains were used for staining and gene expression analysis of embryos:

1. *enok*^*1*^*/CyO, P{GAL4*-*Kr.C}DC3, P{UAS*-*GFP.S65T}DC7* (referred to as *enok*^*1*^).
2. *enok*^*2*^*/CyO, P{GAL4*-*Kr.C}DC3, P{UAS*-*GFP.S65T}DC7* (referred to as *enok*^*2*^).

The following strains were used for polytene staining:

1. *P{w*^+*mC*^*UASp*-*Myc*-*enok}* (generated in this study).
2. *P{Sgs3*-*GAL4.PD}* (BN 6870).

### Genetic analysis

Mutant flies of selected candidates and *w*^*1118*^ were crossed to *Pc* alleles (*Pc*^*1*^ and *Pc*^*XL5*^) at 25 °C. Males in the progeny of these crosses were scored for extra sex combs as described previously [62]. *enok*^*2*^ and *w*^*1118*^ were crossed to *trx* alleles (*trx*^*1*^ and *trx*^*E2*^) at 29 °C. *enok*^*2*^ was also crossed to *w*^*1118*^. The progeny of these crosses were scored for mutant phenotypes.

For analyzing the expression of homeotic genes in vivo, embryos homozygous for *enok*^*1*^ or *enok*^*2*^ mutations were collected at 20 h post-laying from the progeny of a cross between heterozygous mutant alleles, balanced with GFP-marked balancer. Absence of GFP in embryos was an indicator of the required genotype. Same-aged embryos from *w*^*1118*^ were used as control. Embryos were flash frozen in liquid nitrogen and total RNA was isolated using Trizol Reagent (Ambion). The total RNA was treated with TURBO DNase (Ambion) and was used to make cDNA using SuperScript III First-Strand Synthesis System (Invitrogen) following the manufacturer’s instructions. Further, expression levels of *abd A*, *Abd*-*B*, *Dfd*, *Ubx* and *pnt* (with RP49 as normalization control) were quantified with real-time PCR (Applied Biosystems Inc, 7500) using SYBR Green (Applied Biosystems Inc). Primer details can be found in Additional file 5: Table S2.

### Generation of stable cell lines and transgenic flies

To generate vectors expressing tagged proteins, RNA extracted from S2 cells or *w*^*1118*^ embryos was used to make cDNA. Primers designed for Gateway Cloning were used to amplify *enok CDS* from cDNA which was then cloned into *pENTR*-*dTOPO* vector (ThermoFisher Scientific, K240020). LR Clonase reactions (ThermoFisher Scientific, 11791100) were set with DGVC (*Drosophila* Gateway Vector Collection) vectors containing either Myc or FLAG tags to prepare epitope tagged Enok for both cell culture and fly transformation. Primer details can be found in Additional file 5: Table S2.

For the generation of stable cell lines, *pMT*-*FLAG*-*enok* plasmid was transfected into S2 cells using Effectene transfection reagent (Qiagen). Transfected cells were selected using Hygromycin B (final concentration 250 µg/mL). Finally, cells were induced with 500 µM CuSO_4_ for 48 and 72 h and stable cells were confirmed for the expression of our protein by Western blotting with antibody against the FLAG tag.

Fly transformation vector expressing Myc-Enok, under the control of *UASp*, was used to generate transgenic fly lines by injecting *w*^*1118*^ embryos using standard protocol [63]. Transgenic flies were confirmed by Western blotting with α-Myc antibody (Additional file 4: Fig. S3a).

### Immunohistochemistry

Transgenic flies carrying *UAS*-*Myc*-*enok* were crossed with *sgs*-GAL4 driver line. Salivary glands from third instar larvae were isolated and polytene chromosomes were stained with α-TRX and α-Myc using standard protocol [64].

For embryonic staining, stage 15 embryos were dechorionated and GFP-negative embryos were separated under an epifluorescent stereo microscope (Nikon, C-DSS230) and were stained using standard protocol [64]. All images were acquired using the Nikon C2 Confocal Microscope.

### Chromatin immunoprecipitation (ChIP)

ChIP was performed from either stable cell lines induced with CuSO_4_ for 72 h or S2 cells as described previously [46]. Purified ChIP DNA from each reaction was quantified using real-time PCR. Chromatin enrichment as percentage of input was calculated using ΔΔCt method as described previously [65]. For ChIP after knockdown of *enok*, cells were treated with 10 µg/mL of dsRNA for 4–6 days. Knockdown was confirmed by Western blotting. ChIP was then performed with 1 × 10^7^ cells.

Briefly, 3 × 10^7^ cells were fixed at room temperature with 1% formaldehyde for 10 min. Cross-linking was stopped by the addition of glycine to a final concentration of 0.125 M. Cells were washed with 1× PBS and lysed with Buffer A (10 mM Tris, pH 8, 0.25% Triton X-100, 10 mM EDTA, 0.5 mM EGTA) followed by two washes with Buffer B (10 mM Tris, pH 8, 200 mM NaCl, 1 mM EDTA, 0.5 mM EGTA). Cells were sonicated in 300 µl of sonication buffer (10 mM Tris, pH8, 1 mM EDTA, 0.5 mM EGTA) using Bioruptor (Diagenode) at high setting for 15–25 min (30 s on, 30 s off) such that chromatin fragment sizes were between 100 and 500 bp. Sonicated chromatin was centrifuged at 13,000 rpm for 10 min and the cleared chromatin was stored at − 80 °C. Chromatin was diluted with 2× RIPA buffer (20 mM Tris, 2 mM EDTA, 280 mM NaCl, 2%Triton X-100, 0.2% SDS, 0.2% sodium deoxycholate) and precleared by incubating with DYNA beads (Invitrogen) for 2 h at 4 °C with 20 rpm rotation. Precleared chromatin was incubated with the appropriate antibody overnight at 4 °C with 20 rpm rotation. Immunocomplexes were pulled down with DYNA beads. The beads were washed five times with 1x RIPA, once with 1× LiCl Buffer (10 mM Tris, pH 8, 250 mM LiCl, 1 mM EDTA, 0.5% NP-40, 0.5% sodium deoxycholate) and twice with 1× TE (10 mM Tris pH8, 1 mM EDTA). Chromatin was eluted by incubating beads at 65 °C with 500 µl of freshly made elution buffer (0.1 M sodium bicarbonate, 1% SDS) for 15 min. Reverse cross-linking of chromatin was carried out overnight with 5 M NaCl at 65 °C followed by proteinase K treatment for 2 h at 45 °C and reverse cross-linked chromatin was extracted using phenol–chloroform followed by ethanol precipitation. All buffers were supplemented with PMSF, aprotinin, leupeptin and pepstatin protease inhibitors (ThermoFisher Scientific).

## Supplementary information

**Additional file 1: Fig. S1.** (a) *PRE*-Reporter system, with EGFP or Luciferase as read outs, comprised of *Ubx* (*Ultrabithorax*) promoter along with a 1.6 kb *bxd*-*PRE*, flanked by *PBX* (*postbithorax*—embryonic enhancer) and *IDE (Imaginal Disc Enhancer)*. (b) Schematic of working hypothesis for validating PcG/trxG responsive reporter system in *Drosophila* cells. Transient transfections of *PRE*-Reporter constructs (*EGFP/Luc*) along with either overexpression of PcG or depletion of trxG by RNAi would diminish the reporter gene (*EGFP/Luc*) expression and could potentially be used to discover novel players involved in epigenetic cellular memory through genome-wide RNAi screen.

**Additional file 2: Fig. S2.** Analysis of the genome-wide RNAi screens. (a) Schematic of the experimental setup followed for performing genome-wide RNAi screens. (b) Box plots for plate median normalized data for genome screen, replicate 1 (left) and replicate 2 (right). (c) Scatterplots of the plate median corrected intensity values for F.Luc against the plate median corrected intensity values for R.Luc for replicate 1 (left) and replicate 2 (right) genome-wide RNAi screen. Cut-off values (dashed line) were set using *trx* and *ash1* Z-scores. Knockdown of genes that affected both F.Luc and R.Luc were removed from further analysis and were masked (shown in grey). Positive controls are shown in red (*trx*), brown (*ash1*) and blue (*F.Luc*) and negative controls are shown in green (*LacZ*) and orange (*GFP*). Knockdown of *Thread* (*Diap*-*1*) gene was used as a control for RNAi efficiency in genome-wide RNAi screen.

**Additional file 3: Table S1.** List of candidates generated from the genome-wide RNAi screen along with their average Z-scores.

**Additional file 4: Fig. S3.** Enok colocalizes with PC on polytene chromosomes. (a) Confirmation of transgenic flies expressing Myc-tagged Enok on Western blot with α-Myc antibodies. Myc-Enok can be specifically seen in transgenic flies whereas it is absent in *w*^*1118*^ flies. (b–e) Polytene chromosomes from third instar larvae of transgenic flies expressing Myc-tagged Enok stained with α-Myc (c) and α-PC (d) antibodies. Enok and PC were seen to co-localize at several loci in merge (e). (f) Western blot analysis of *Drosophila* S2 stable cell line, expressing FLAG-tagged Enok (FH-Enok) under copper inducible promoter. 48 and 72 h after induction (I) cells show a marked increase in Enok protein as compared to uninduced (U) cells. (g) Knockdown of *enok* shows drastic decrease in amount of Enok protein on Western blot when compared to cells treated with *LacZ* dsRNA. Tubulin levels remained the same. (h) Cells treated with dsRNA against *enok* were analyzed for the expression level of *trx* mRNA. As compared to *LacZ* dsRNA treated control cells, there was no significant change in the amount of *trx* mRNA expressed in *enok* depleted cells. (i, j) Knockdown of *enok* had no effect on the occupancy of TRX (i) or levels of H3K27ac (j) at PcG/trxG target sites. (k) Knockdown of *enok* shows a drastic reduction in global levels of H3K23ac when compared to cells treated with *LacZ* dsRNA. There was no effect on total levels of histone H3 which was used as a control. (l, m) Knockdown of *enok* has no effect on the occupancy of E(z) (l) or levels of H3K27me3 (m) at PcG/trxG target sites. Experiments were performed in triplicates and individual student *t*-tests were performed to analyze the results.

**Additional file 5: Table S2.** List of primers used in this study.

## Data Availability

All data generated and analyzed in the study are available in the main or additional files provided.

## Abbreviations

Pc: Polycomb
trx: trithorax
PcG: Polycomb group of proteins
trxG: trithorax group of proteins
PRC1: Polycomb Repressive Complex 1
PRC2: Polycomb Repressive Complex 2
PRE: Polycomb Response Element
TRE: Trithorax Response Element
Enok: enoki mushroom
H2AK118ub1: histone H2A lysine 118 mono-ubiquitination
H3K4me3: histone H3 lysine 4 trimethylation
H3K27ac: histone H3 lysine 27 acetylation
H3K27me3: histone H3 lysine 27 trimethylation
Ubx: ultrabithorax
PBX: postbithorax
IDE: Imaginal Disc Enhancer
EGFP: Enhanced Green Fluorescent Protein
RFP: red fluorescent protein
F.Luc: Firefly Luciferase
R.Luc: Renilla Luciferase
dsRNA: double-stranded RNA
HD2: Heidelberg 2
E(z): Enhancer of Zeste
ash1: absent small or homeotic discs1
brm: brahma
mor: moira
abd-A: abdominal-A
Abd-B: Abdominal-B
Dfd: Deformed
pnt: pointed
psq: pipsqueak
pnr: pannier
disco: disconnected
Pol-II: RNA polymerase II
TSS: transcription start site
IR: intergenic region
