# Supplementary figures and images for: Genome-wide RNAi screen in Drosophila reveals Enok as a novel trithorax group regulator
Source: Epigenetics Chromatin. 2019 Sep 23;12:55. doi: 10.1186/s13072-019-0301-x (PMC6757429; doi:10.1186/s13072-019-0301-x)

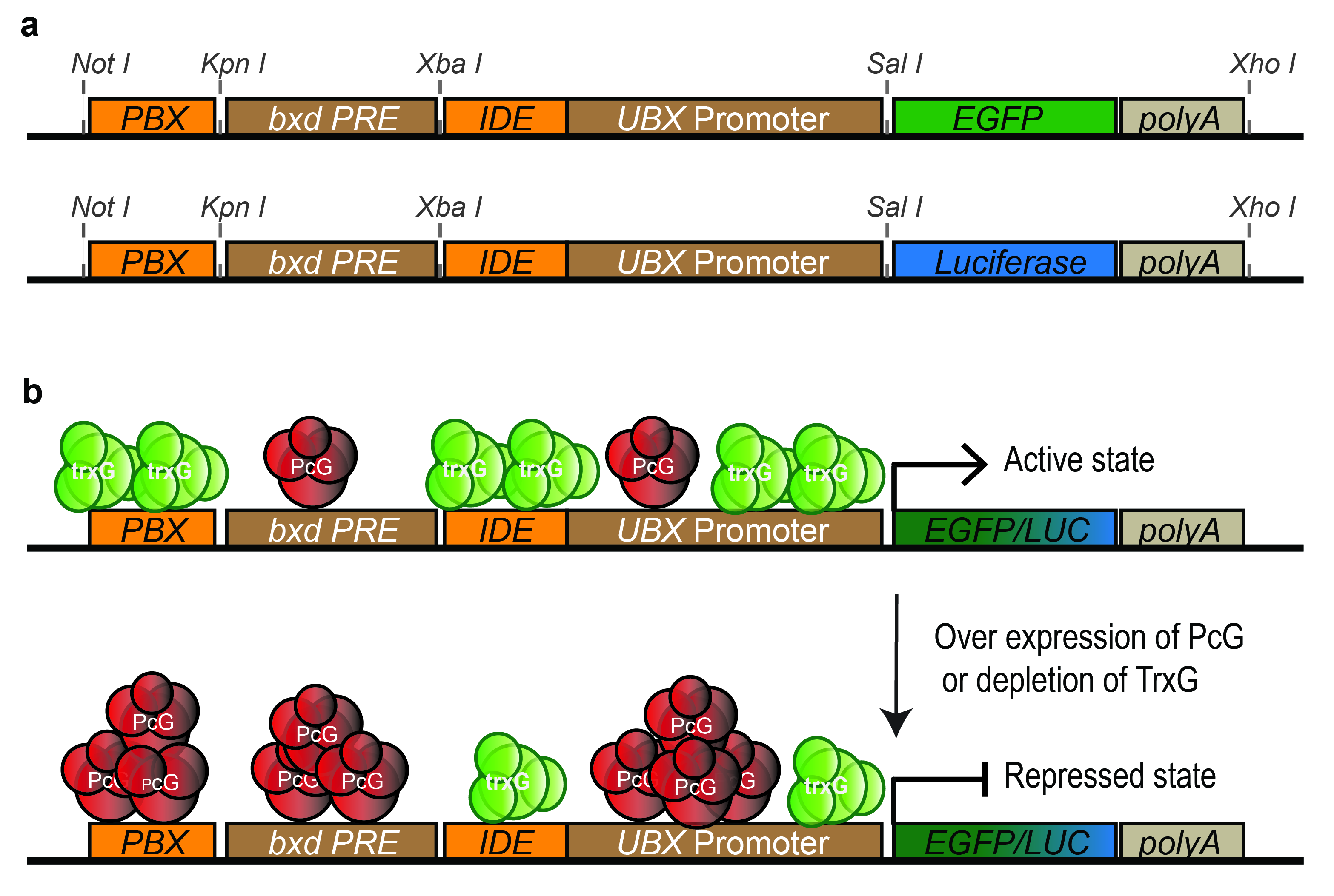

Supplement: Supplementary file 1 — Additional file 1: Fig. S1. (a) PRE-Reporter system, with EGFP or Luciferase as read outs, comprised of Ubx (Ultrabithorax) promoter along with a 1.6 kb bxd-PRE, flanked by PBX (postbithorax—embryonic enhancer) and IDE (Imaginal Disc Enhancer). (b) Schematic of working hypothesis for validating PcG/trxG responsive reporter system in Drosophila cells. Transient transfections of PRE-Reporter constructs (EGFP/Luc) along with either overexpression of PcG or depletion of trxG by RNAi would diminish the reporter gene (EGFP/Luc) expression and could potentially be used to discover novel players involved in epigenetic cellular memory through genome-wide RNAi screen. [file 13072_2019_301_MOESM1_ESM.tif]

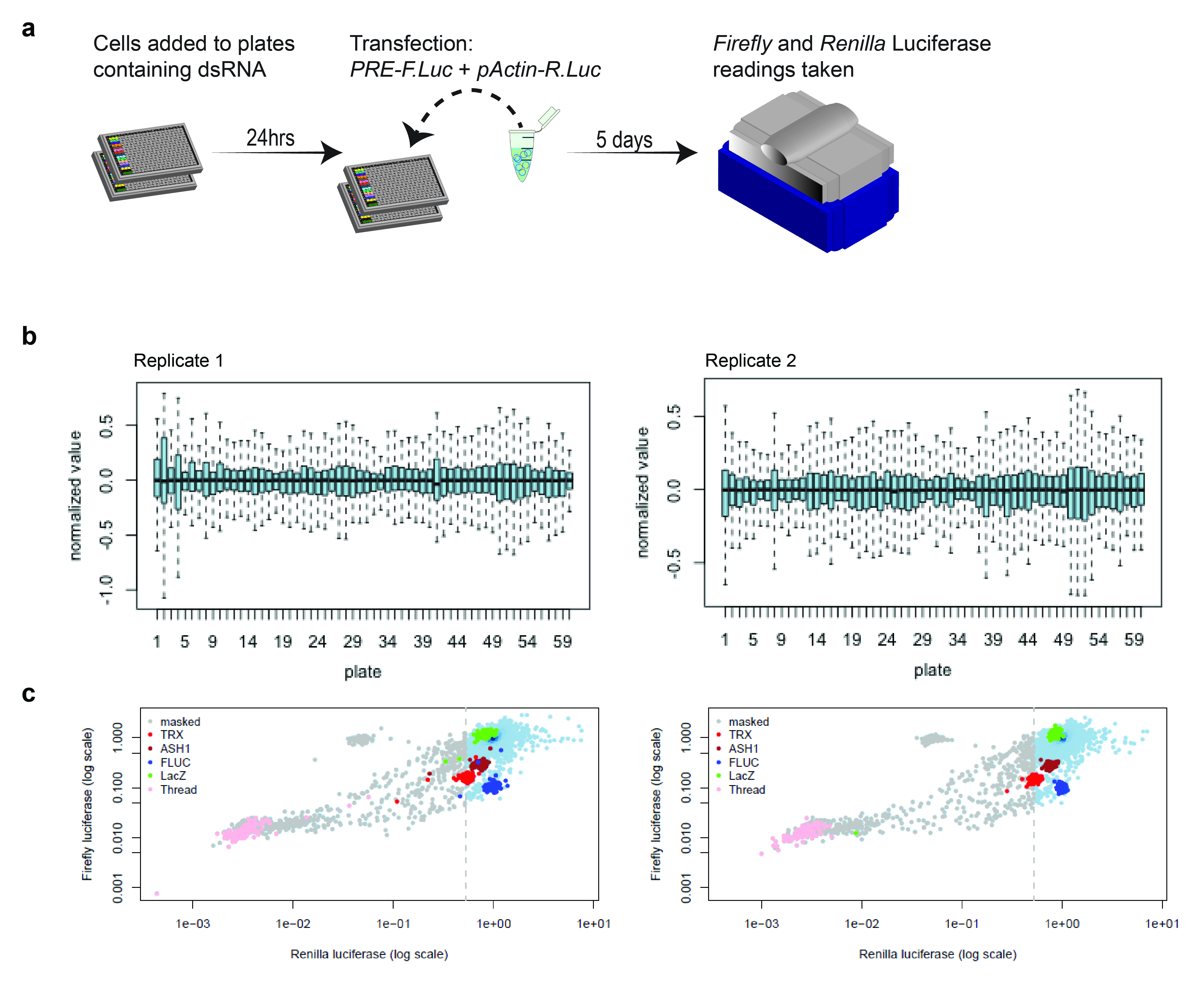

Supplement: Supplementary file 2 — Additional file 2: Fig. S2. Analysis of the genome-wide RNAi screens. (a) Schematic of the experimental setup followed for performing genome-wide RNAi screens. (b) Box plots for plate median normalized data for genome screen, replicate 1 (left) and replicate 2 (right). (c) Scatterplots of the plate median corrected intensity values for F.Luc against the plate median corrected intensity values for R.Luc for replicate 1 (left) and replicate 2 (right) genome-wide RNAi screen. Cut-off values (dashed line) were set using trx and ash1 Z-scores. Knockdown of genes that affected both F.Luc and R.Luc were removed from further analysis and were masked (shown in grey). Positive controls are shown in red (trx), brown (ash1) and blue (F.Luc) and negative controls are shown in green (LacZ) and orange (GFP). Knockdown of Thread (Diap-1) gene was used as a control for RNAi efficiency in genome-wide RNAi screen. [file 13072_2019_301_MOESM2_ESM.tif]

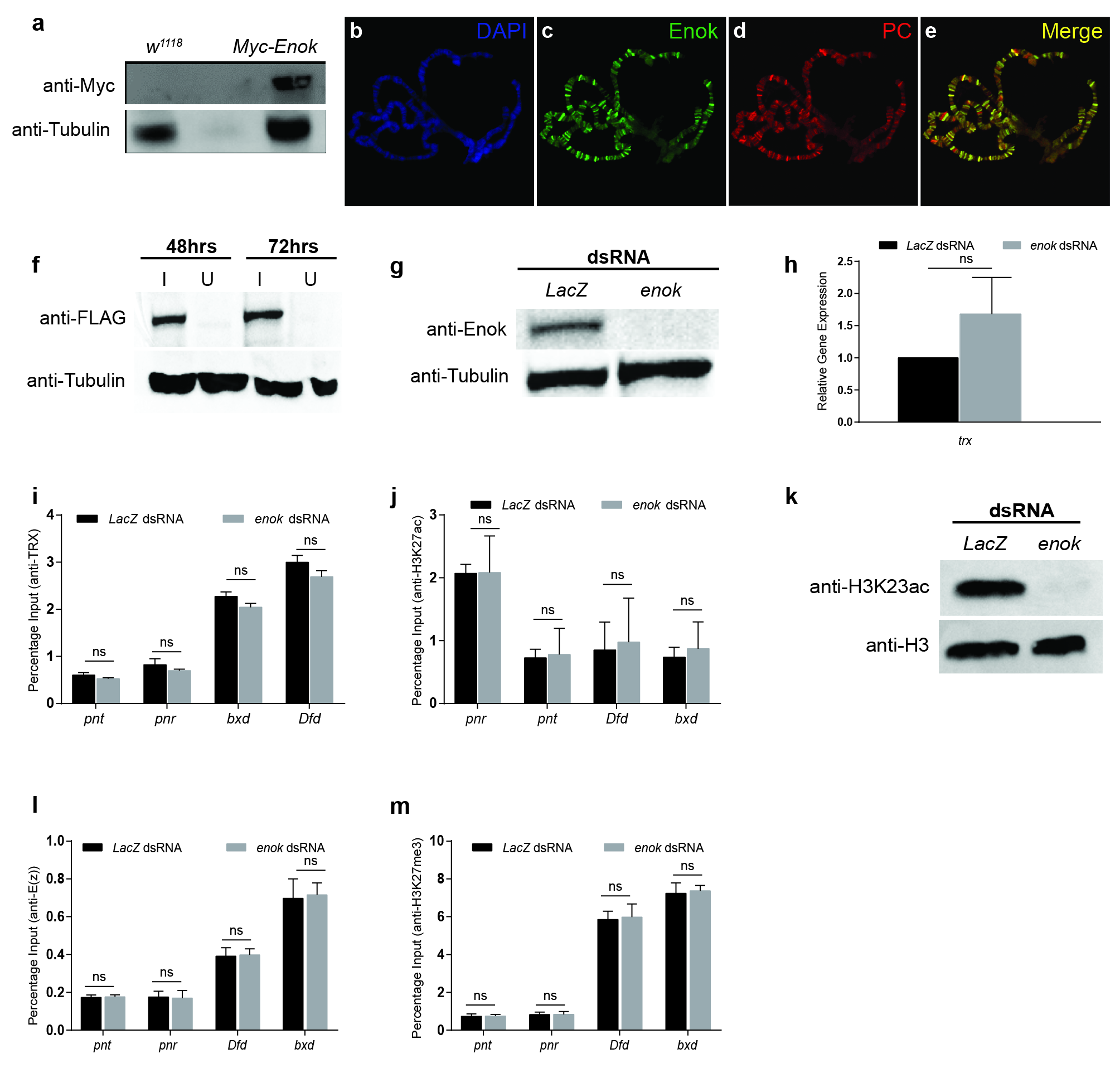

Supplement: Supplementary file 4 — Additional file 4: Fig. S3. Enok colocalizes with PC on polytene chromosomes. (a) Confirmation of transgenic flies expressing Myc-tagged Enok on Western blot with α-Myc antibodies. Myc-Enok can be specifically seen in transgenic flies whereas it is absent in w1118 flies. (b–e) Polytene chromosomes from third instar larvae of transgenic flies expressing Myc-tagged Enok stained with α-Myc (c) and α-PC (d) antibodies. Enok and PC were seen to co-localize at several loci in merge (e). (f) Western blot analysis of Drosophila S2 stable cell line, expressing FLAG-tagged Enok (FH-Enok) under copper inducible promoter. 48 and 72 h after induction (I) cells show a marked increase in Enok protein as compared to uninduced (U) cells. (g) Knockdown of enok shows drastic decrease in amount of Enok protein on Western blot when compared to cells treated with LacZ dsRNA. Tubulin levels remained the same. (h) Cells treated with dsRNA against enok were analyzed for the expression level of trx mRNA. As compared to LacZ dsRNA treated control cells, there was no significant change in the amount of trx mRNA expressed in enok depleted cells. (i, j) Knockdown of enok had no effect on the occupancy of TRX (i) or levels of H3K27ac (j) at PcG/trxG target sites. (k) Knockdown of enok shows a drastic reduction in global levels of H3K23ac when compared to cells treated with LacZ dsRNA. There was no effect on total levels of histone H3 which was used as a control. (l, m) Knockdown of enok has no effect on the occupancy of E(z) (l) or levels of H3K27me3 (m) at PcG/trxG target sites. Experiments were performed in triplicates and individual student t-tests were performed to analyze the results. [file 13072_2019_301_MOESM4_ESM.tif]
