# Supplementary material for: Genome-wide RNAi screen in Drosophila reveals Enok as a novel trithorax group regulator
Source: Epigenetics Chromatin. 2019 Sep 23;12:55. doi: 10.1186/s13072-019-0301-x (PMC6757429; doi:10.1186/s13072-019-0301-x)
Supplement: Supplementary file 5 — Additional file 5: Table S2. List of primers used in this study. [file 13072_2019_301_MOESM5_ESM.docx]

**Table S2: List of primers used in this study**

| **Sr No.** | **Primer Name** | **Sequence** | **Purpose** |
| --- | --- | --- | --- |
| 1 | EGFP-Sal1-F | ACGCGTCGACGGATGGTGAGCAAGGGCGA | Cloning of Reporters into fly vector |
| 2 | EGFP-Sal1-R | ACGCGTCGACTTACTTGTACAGCTCGTCCATG |  |
| 3 | Luc-Sal1-F | GCAGGTCGACGGATGGAAGACGCCAA |  |
| 4 | Luc-Sal1-R | CTTAGTCGACTTACACGGCGATCTTTCCG |  |
| 5 | HN1 | AGCTTGCATGCGCGGCCGCTATGCG | Linkers for *pCoBlast* |
| 6 | HN2 | CGCATAGCGGCCGCGCATGCA |  |
| 7 | SN1 | TCGACCTGCAGGCGGCCGCTATGCG |  |
| 8 | SN2 | CGCATAGCGGCCGCCTGCAGG |  |
| 9 | Xho1-Luc-F | CCGCTCGAGGGATGGAAGACGCCAA | Cloning of Reporters into *pCoBlast* |
| 10 | Xho1-EGFP-F | CCGCTCGAGGGATGGTGAGCAAGGGCGA |  |
| 11 | Xho1-hsp poly A | CCGCTCGAGGAGCTCTCCTGACCGTCC |  |
|  |  |  |  |
| 12 | DRSC04096 F | **TAATACGACTCACTATAGGGAGA**CGTCTAATGAGGCAAAGAAAC | Enok dsRNA |
| 13 | DRSC04096 R | **TAATACGACTCACTATAGGGAGA**CCGTTTTTGCCACTTTAACC |  |
| 14 | T7-LacZ F | **TAATACGACTCACTATAGGGAGA**GGAAGATCAGGATATGTGG | LacZ dsRNA |
| 15 | T7-LacZ R | **TAATACGACTCACTATAGGGAGA**CTTCATCAGCAGGATATCC |  |
| 16 | DRSC24966 F | **TAATACGACTCACTATAGGGAGA**GAAGCCATAAACACAACGCC | PC dsRNA |
| 17 | DRSC24966 R | **TAATACGACTCACTATAGGGAGA**ACATTTGTTTGGGTCGAAGC |  |
|  |  |  |  |
| 18 | Enok-F1 | CACCATGATGAGGGAATCGGCGCA | Cloning of full length *enok* in pENTR |
| 19 | Enok-R1 | TGGCTGCGGGGGACGTGGCA |  |
| 20 | Enok-F2 | CACCGTACTGGCGATGGCCTA |  |
| 21 | Enok-R2 | TCTGCACTGAGGCCACTGGT |  |
| 22 | Enok-F3 | CACCATTGCAGCACGTACAT |  |
| 23 | Enok-R3 (NS) | TTATCTGCGAATAGAACCGT |  |
|  |  |  |  |
| 24 | bxd-s-low | GCACTTAAAACGGCCATTACGAA | Primers used for analysis of ChIP |
| 25 | bxd-s-up | GACGTGCGTAAGAGCGAGATACAG |  |
| 26 | Dfd F | AACTCTCCGTGCGAGCGAAC |  |
| 27 | Dfd R | ATGCTCCCTCTCAGTCGCGCT |  |
| 28 | disco_F | GTTTCGTTGGGTTGACACATG |  |
| 29 | disco_R | CATTGCCATTTCACTCTCGTTG |  |
| 30 | iab7-4-low | AGCTTTTGCCACTCGTCCTGTT |  |
| 31 | iab7-4-up | AGCAGAGCTGTGCCATTGTTT |  |
| 32 | Intergenic Region F | CCGAACATGAGACATGGAAAA |  |
| 33 | Intergenic Region R | AAAGTGCCGACAATGCAGTTA |  |
| 34 | pnr_TSS_F | TCTCTTGCTCTTTCGCTCAC |  |
| 35 | pnr_TSS_R | GTTTTCCATACGCACTCACAC |  |
| 36 | pnt_TSS_F | TCATTCCAGCGATCAAGTAAAA |  |
| 37 | pnt_TSS_R | TCTTTCTCTCCGCTGCTAAGAT |  |
| 38 | psq_TSS_F | ATAAGGCGATGCCACCTAGTTA |  |
| 39 | psq_TSS_R | AATGTAGCAAAAGGTGCTCAAAG |  |
| 40 | pnr_Gene Body_F | CCCAGTGGCGACTCATTAGA |  |
| 41 | pnr_Gene Body_R | AGATTGTGTAGTGGTCGAGCA |  |
| 42 | pnt_Gene Body_F | AGTCCATCAAGATTGTGCGC |  |
| 43 | pnt_Gene Body_R | TAAAATAGCGCCCCTTCGTG |  |
|  |  |  |  |
| 44 | Act57BF | TGTGTGACGATGAAGTTGCTGC | Primers used for qPCR Analysis |
| 45 | Act57BR | ATCACCGACGTACGAGTCCTT |  |
| 46 | AbdAF | TGACGCTTACAGACTGGATGG |  |
| 47 | AbdAR | CGCGCCTGTTCATTTATTTCC |  |
| 48 | AbdB ex1.1 F | CAACTACCGAACTAAGCTGC |  |
| 49 | AbdB ex1.2 R | CACAATGAGGAGCAAGGATG |  |
| 50 | DfdF | CGATGGCGAACGGATCATCTA |  |
| 51 | DfdR | GCGTCAGGTAGCGGTTGTAGTGG |  |
| 52 | pnr_E2F | GATGGAACCGGACACTATCTGT |  |
| 53 | pnr_E3R | AAAGTGTGGTGGTCCGAGTG |  |
| 54 | pnt_E5F | ACCTGGACCCCTTCTACAAGAT |  |
| 55 | pnt_E5R | CCGTGCACAATATCCTCATTT |  |
| 56 | rp49 F | GACGCTTCAAGGGACAGTATCTG |  |
| 57 | rp49 R | AAACGCGGTTCTGCATGAG |  |
| 58 | Ubx F | ATGAACTCGTACTTTGAACAGGC |  |
| 59 | Ubx R | CCAGCGAGAGAGGGAATCC |  |
